# Epidural Catheter in a Child with Metastatic Rhabdomyosarcoma

**DOI:** 10.7759/cureus.2880

**Published:** 2018-06-26

**Authors:** Lisgelia Santana

**Affiliations:** 1 Anesthesiology and Pain Management, Nemours Children's Hospital, Orlando, USA

**Keywords:** pain medicine, hospice/palliative medicine, pediatric cancer, pediatric pain, epidural catheter, tunnel catheter

## Abstract

Pain and symptom management is a cornerstone of palliative and hospice medicine. The aim of this article is to educate clinicians about the uncommon causes of bleeding from an epidural catheter for hospice pain management. A case of a 12-year-old female with progressive metastatic rhabdomyosarcoma-left forearm primary who had exhausted all treatment options is reported. She had a very significant cancer-related pain, which was not amenable to hospice management at home. A tunneled epidural catheter was placed so that she could receive better pain management at home as her life expectancy was very short. The patient had massive bleeding coming from the tunnel site developing big clots around dressings on the third day after the catheter placement. All methods for stopping the bleeding were employed but it stopped only after the epidural catheter was removed. In conclusion, the development of pain management strategy using multidisciplinary inputs with appropriate, timely use of interventional pain management techniques provides satisfactory pain relief for these patients and reduces distress in patients and relatives during this difficult period. Multiple approaches exist for pain management; however, systemic medications sometimes cause additional side effects (nausea, vomiting, constipation, drowsiness, respiratory depression). Unfortunately, some interventional procedures may also have side effects (bleeding, infection, ineffectiveness).

## Introduction

The management of pain due to cancer is challenging and it often requires invasive therapy in addition to medication management. Clinicians may avoid continuous pain blocks in pediatric cancer patients at the end of life for fear of complications or for fear of interfering with the desired location of death. Although risks associated with epidural drug delivery are low, some common complications include dislodgement, kinking, or fracture of the catheter, bleeding, neurological injury, and infection. A study conducted in 1992 consisted of 142 patients with refractory cancer, who were getting an intrathecal catheter for pain management. They showed that the least frequent complication was bleeding in the tunnel site (0.7%) [1]. Thorough fixation of the epidural catheter by tunneling and suturing decreases the incidence and extent of dislocation and potentially even that of bacterial contamination [2-3]. A report published in the year 2000 speculated that the catheter movement within the skin may potentially contribute to bacterial contamination, which is possibly linked to catheter-related infective complications with colonization rates as high as 12% [4]. A tunneled catheter will decrease the risk of dislocation and contamination. 

The present case shows that despite coagulopathy being the most obvious cause of massive bleeding, tunneling by itself may cause life-greatening bleeding in a cancer patient. 

## Case presentation

The present case is a 12-year-old girl with progressive metastatic rhabdomyosarcoma-left forearm primary. Metastasis was identified on the right lumbar paraspinal muscles, left femur, and left jaw. Multiple magnetic resonace imaging (MRI) and computed tomography (CT) scans showed no involvement of the spinal cord. She did not receive more chemotherapy or radiation therapy. She had a very significant cancer-related pain, especially in her lower extremities and jaw, which was not amenable to hospice management at home with hydromorphone patient controlled analgesia (PCA). She was admitted to the hospital due to poor pain control and the pain team was consulted for better pain management options. Her life expectancy is very short and a decision had to be made to place a tunneled epidural catheter to send her home. Due to significant debilitating headaches and the possibility of cerebral spinal fluid leak and postdural puncture headaches during an intrathecal catheter placement, this therapy was not considered as the first option. Her parents are hoping that with the epidural she will be less sedated than with the hydromorphone PCA. The patient received a transfusion of fresh frozen plasma to get a normal prothrombin time (PT), partial thromboplastin time (PTT), and international normalized ratio (INR). She also needed a platelet transfusion, which brought her level to 109,000 during the morning time of the procedure. Her initial hemoglobin level is 9.1 g/dL on the day of the procedure. She was brought to the operating room for sedation. The epidural catheter is placed without complication; the catheter tip is confirmed at the L2-L3 level with fluoroscopy and 1 millimeter (ml) of contrast dye (Omnipaque 300, GE Healthcare, Cork, Ireland) and subcutaneously tunneled completely under the left paraspinal muscles with a Touhy 18-gauge 3.5 inches needle (B. Braun Medical, Inc., PA, USA). The needle was removed carefully keeping the catheter in place, and no signs of bleeding were observed after the needle was removed. The patient reported analgesia from epidural and PCA dose was weaned down. Overnight she started leaking from the epidural and tunneled sites; coagulation parameters were normal. A neuro check was normal after stopping the epidural infusion for two hours. On the second day, the bleeding was more pronounced, her platelets were 96,000 INR 1.2, and the surgical hemostat was applied with pressure dressings. The neuro checks continued to rule out epidural hematoma and spinal cord injury. Aspiration of the epidural catheter was bloodless, and a test dose of 3 mm of lidocaine 1.5% with epinephrine 1:200,000 was negative, ruling out migration to a vessel. On the third day, the patient had massive bleeding, which came from the tunnel site developing big clots around the dressings. Aspiration of the epidural catheter was bloodless, and a test dose of 3 mm of lidocaine 1.5% with epinephrine 1:200,000 was negative. Thrombin, Stat Seal disc (Biolife, LLC, Sarasota, FL, USA), Quick Clot (Z-MEDICA, LLC, Wallingford, CT, USA), and all hemostatic agents were applied, without any success. The patient was transfused with platelets (her count was 76,000), fresh frozen plasma, and packed red blood cells because at this point, her hemoglobin level was 5.4 g/dL. Bleeding resolved after removing the epidural catheter. 

## Discussion

Bleeding after epidural catheter placement is rarely seen; usually it can happen because of coagulopathies, traumatic needle insertion, and spontaneous bleeding. In a meta-analysis of 613 case reports from 1826 to 1996, the primary etiology of epidural hematoma was idiopathic, followed by coagulopathies [5]. In 2010, Anghelescu et al. published a review of 10 patients with 14 catheters (11 epidural, three peripheral nerve catheters) for a range of 3-81 days with excellent pain control and no bleeding, infections, or neurological complications [6]. Dislocation of epidural catheters may cause early termination of postoperative regional analgesia. Moreover, Gogarten et al. [7] and Horlocker et al. [8] reported that accidental removal shortly after anticoagulant administration, or in patients with coagulopathies may increase the risk of epidural hematoma and neurologic complications.

## Conclusions

Bleeding epidural catheters are rare and they are usually related to coagulopathies, especially in cancer patients. In the present case, the anesthesiologist made sure that the patient’s coagulations were normal for insertion and removal of the catheter. Cancer patients might have more friable tissues related to malignancy or poor nutrition, and tunneling can be traumatic and can cause profuse bleeding. In addition, physicians need to consider new metastasis on the injection sites. In the present case patient was not getting more imaging studies due to the end stage disease, making it difficult to identify new tumors. 
